# Correction: Network analysis reveals abnormal functional brain circuitry in anxious dogs

**DOI:** 10.1371/journal.pone.0320459

**Published:** 2025-03-17

**Authors:** Yangfeng Xu, Emma Christiaen, Sara De Witte, Qinyuan Chen, Kathelijne Peremans, Jimmy H. Saunders, Christian Vanhove, Chris Baeken

S1 Text is incorrect. Please view the correct S1 Text below.

## Supporting information

S1 Text Protocol of the Faculty of Veterinary Medicine at Ghent University for monitoring the welfare of dogs kept and used for research purposes. (PDF)

In the Animal subsection of Materials and methods, there is an error in the eighth sentence of the first paragraph. The correct sentence is: Furthermore, all these dogs displayed normal behavior, evaluated by both veterinarians involved and care takers of the dogs regularly (Protocol of the Faculty of Veterinary Medicine at Ghent University for monitoring the welfare of dogs kept and used for research purposes) (Details in S1 Text).
