# Supplementary material for: Network analysis reveals abnormal functional brain circuitry in anxious dogs
Source: PLoS One. 2023 Mar 15;18(3):e0282087. doi: 10.1371/journal.pone.0282087 (PMC10016658; doi:10.1371/journal.pone.0282087)
Supplement: S1 Text — (PDF) [file pone.0282087.s001.pdf]

Monitoring of welfare in dogs kept and used for research purposes

The dog welfare monitoring protocol consists of three main sections:

A. Health monitoring (p.1)
B. Spontaneous behaviour in the kennel (p.2)
C. Behavioural responses in different contexts (p.3-9)

All scores for this protocol can be entered in the template (per dog) that has been provided.

A. Health monitoring

1) Routine health checks

This check occurs at least once per year. Records of this evaluation must be kept for each dog.

Particular attention to possibly painful conditions using the following nine items: eyes, ears, teeth/oral cavity/nose, chest, abdomen, feet, skin, gait, pulse and respiration.

Scoring per item:

- 0 = no obvious problem
- 1 = problem that requires further monitoring, but no treatment
- 2 = problem that requires one-time treatment
- 3 = problem that requires repeated (daily/weekly) treatment.

Bloodwork is also carried out once per year.

2) Increased frequency of health checks in relation to experimental interventions and/or lab practicals.

A health check is conducted before an experiment or series of lab practicals starts, according to the procedure described in A.1). An exception to this rule is when the lab practical consists of a clinical examination.

Records must be kept for each dog, also when the examination occurs during the lab practical.

3) Increased frequency of health checks in case of score 1, 2 or 3 in A.1) or A.2).

The frequency must be determined as needed for the particular problem. Records must be kept separately, by ID, for the respective dogs.

B. Spontaneous behaviour in the kennel

1) Observations of spontaneous behaviour

These observations are carried out once per year. It is expected that the emotion of the dog in the kennels is not prone to much change.

Record 60 minutes of video **during a quiet time** in the kennel (no cleaning, minimum passage of people in the building); choose a time that is feasible for each subsequent evaluation, so that dogs are observed at the same time during the day (to avoid effects of circadian rhythm). Point the camera in such a way that it covers the exit door to the outside run and as many of the rest of the kennels in which a group of dogs reside.

Analyse **minutes 30 to 40** (= 600 seconds) and **50 to 60** (= 600 seconds) of the video for the following:

a) **Occurrence (in seconds) of lying down being alert (head up), lying down with head down and eyes open, lying down with head down and eyes closed, being upright, walking around, and time that the dog is "out of sight" (you cannot see the dog or see what it is doing).**
You can use the additional template that is provided for this.

These data are then processed, both as absolute values per dog and relative to other dogs in the kennel

Absolute: For each dog, calculate the proportion for each behaviour, except "out of sight". The formula = [duration of the behaviour in seconds, divided by (600 seconds minus duration of "out of sight" in seconds)] to the time that the dog is lying down relaxed, lying down alert, being upright/walking and paying attention to environment, being upright/walking and paying attention to other dogs in the room.

Scoring:

- Score 0 = more than 50 % of the time lying down relaxed
- Score 1: More than 50 % of the time being upright or walking around paying attention to other dogs
- Score 2 = more than 50 % of the time being upright or walking around paying attention to environment.

Relative:
From the template, determine per dog how the total duration (in seconds) of lying down with head down (eyes open or closed), compares to the other dogs in the kennel.

Scoring:

- Score 0 = lying down more than at least half of the other dogs
- Score 1 = lying down less than at least half of the other dogs

b) **When dogs are lying down, observe for each dog the <u>response to external disturbance</u> in relation to other dogs (gets up first, last or not; lies down again first, last or not).**
   - **If the dog is not lying down in those 20 minutes or there is no obvious disturbance, check the rest of the video (the entire 60 minutes) to see if you can find such an event.**
   - **If you cannot find a time on the video when the dog is lying down at all, this needs to be noted and monitored across assessment sessions since it could indicate a physical or social problem (no score is given).**
   - **If the dog is lying down, but this does not occur at the same time as when a disturbance happens, it is noted as such (no score given).**

   Scoring:
   - Score 0 = does not get up
   - Score 1 = gets up later than half of the other dogs + lies down quicker than half of the other dogs, or gets up quicker than half of the other dogs + lies down quicker than half of the other dogs
   - Score 2 = gets up quicker than half of the other dogs and lies down later than half of the other dogs or gets up later than half of the other dogs + lies down later than half of the other dogs.
   - Score 3= never lies down

c) **<u>Proximity to other dogs. Scan through the video again and see if the dog is consistently lying near other dogs or away from them and/or it avoids some of the other dogs while awake.</u>**
   Scoring:
   - Score 0 = no avoidance of any other dog while awake / or sleeping near all other dogs (other dogs as one group)
   - Score 1 = One observed avoidance of any other dog while awake or sleeping near one or two dogs, but clearly separated from the other dogs
   - Score 2 = Repeated avoidance observed while awake or sleeping entirely separately, away from all other dogs

Scores of at least 2 should be considered worrisome. Monitor the evolution of each of the scores over time.

2) Additional behaviour observations

In cases of increasing severity of scores in this section (B) or worrisome observations in the next section (C), the observation of spontaneous behaviour in the kennel environment will be executed in between the planned yearly observations.

3) Consulting a behaviour specialist

In case of additional behaviour observations as described in B.2), and if scores of 2 or 3 on any of the items in B.1) for a spontaneous behaviour observation session, contact a behaviour specialist for further advice.

C. Behavioural responses in different contexts

These observations are carried out before and after an experiment in case the duration of the experiment is less than 5 months. If the experiment lasts 5 or 6 months, the observations are carried out before, after and once in the middle. If an experiment lasts longer than 6 months, the observations are carried out before, after and at least once every 3 months during the experiment.

All the items are to be carried out consecutively, as one session, by one person. This person is referred to as "the tester". It is a person person carrying out experimental handling or lab practicals on the dog – this should not be a person who is unfamiliar to the dogs or with whom the dogs only have pleasant experiences). The tester carries out all the actions with the dogs as described below. He or she can observe the behaviour and write down the scores, although it will be easier if a second person (The Observer) is present to observe the behaviour, assist with test 9 (attention test – holds the leash), and write down the scores. One can also choose to record the tests on video and score them later.

If a certain location requires a different sequence of tests, that sequence remains the same for all future tests.

In between each test, allow for 1 minute of recovery. During this time, do not interact with the dog (no speaking to, no touching). When using video, continue to film the dog during the recovery time, so that the video for all tests consists of one file to store/analyse.

Data for this section are collected without assigning critical scores. However, deterioration (increase) of scores over three time points should be monitored and/or discussed with a behaviour specialist.

1) **Approaching the dog in the kennel** (adapted from Valsecchi et al. 2011)

   The tester quietly approaches the kennel with a neutral posture, avoiding direct eye contact, and stops about 20 centimetres from the fence, facing the kennel. Observe the initial response by the dog (10 seconds).

   Scoring:
   - Score 0 = Neutral or friendly, dog can be calm or excited
   - Score 1 = Fearful / anxious
   - Score 2 = Threatening

   <u>Note: Definitions here are as follows:</u>
   - "neutral" means dog holds still, showing neither threatening nor friendly behaviours
   - "friendly" means tail held high and wagging, nonaggressive barking, and/or approach seeking contact with the tester without backing away
   - "fearful/anxious" can have various presentations: a crouched posture, including ears and tail held low, avoiding eye gaze. Tail may be wagging, but is held low. Possibly shaking or whimpering. It is also possible, however, that the dog shows great excitation and jumps up against the tester, or is running back and forth, excitedly and/or in an erratic manner. Fearful/anxious may also include backing away from the front of the cage, in an attempt to avoid the tester.
   - "threatening" means barking, growling, lunching towards the mesh, stiff posture, piloerection etc.

2) Removing dog from kennel (unreferenced)

The tester opens the door and invites the dog to come out of the kennel (possibly stopping other dogs from exiting as well).

Scoring:

- Score 0 = dog exits the kennel without hesitation
- Score 1 = dog hesitates briefly, but exits the kennel on its own
- Score 2 = dog shies away and tester has to go in the kennel to get the dog

3) Putting on collar/harness and leash (adapted from Valsecchi et al. 2011)

A collar or harness, which is attached to a leash, is put onto the dog by the tester. Scores refer to how easily the tester succeeds in this operation. If the dog is very excited, the tester can try to calm it by talking in gentle tones.

Scoring:

- Score 0 = the dog is confident and it is easy to put the collar on
- Score 1 = the dog is hesitant/fearful, but it is easy to put the collar on
- Score 2 = the dog is excited and unmanageable, or reluctant/fearful and it is laborious to put the collar on
- Score 3 = the dog reacts aggressively and it is impossible to put the collar on

4) Walking in the animal house hallway (scoring adapted from Valsecchi et al. 2011)

The dog is walked out of the kennel room into the hallway and walked through the animal house hallway. There should not be other people or dogs present in the hallway.

Scoring:

- Score 0 = the dog walks smoothly through the corridor (relaxed posture, tail base is not tucked), seems interested in the environment
- Score 1 = the dog is hesitant (may have lower body posture and tail base is possibly tucked), but when the handler walks, the dog follows and it does not stop intermittently
- Score 2 = The dog walks with the handler, but stops intermittently (by sitting or lying down without sniffing) during walking
- Score 3 = The dog is clearly scared (flattens itself when walking or stopping) and/or wants to turn back.

5) **Walking outside** (scoring adapted from Valsecchi et al. 2011)

The dog and tester exit the animal house building and walk outside along the green area. Behaviour is scored when no disturbances occur (disturbance = car passing, other dog…). If a disturbance did occur, wait a few minutes before scoring the behaviour.

Scoring:

- Score 0 = the dog walks smoothly (relaxed posture, tail base is not tucked), seems interested in the environment
- Score 1 = the dog is hesitant (may have lower body posture and tail base is possibly tucked), but when the handler walks, the dog follows and it does not stop intermittently
- Score 2 = The dog walks with the handler, but stops intermittently (by sitting or lying down without sniffing) during walking
- Score 3 = The dog is clearly scared (flattens itself when walking or stopping) and/or wants to turn back. Stop the test and return the dog to its kennel.

6) **Walking toward room familiar to the dog for experimental interventions and/or lab practicals** (scoring adapted from Valsecchi et al. 2011)

Enter the clinic hallway or other area while going towards a room known to the dog for experimental interventions and/or lab practicals.

Scoring:

- Score 0 = the dog walks smoothly through the corridor (relaxed posture, tail base is not tucked), seems interested in the environment
- Score 1 = the dog is hesitant (may have lower body posture and tail base is possibly tucked), but when the handler walks, the dog follows and it does not stop intermittently
- Score 2 = The dog walks with the handler, but stops intermittently (by sitting or lying down without sniffing) during walking
- Score 3 = The dog is clearly scared (flattens itself when walking or stopping) and/or wants to turn back

7) **Walking into a room familiar to the dog for experimental interventions and/or lab practicals** (scoring adapted from Valsecchi et al. 2011)

The tester now walks into a room that the dog is familiar with in the context of experimental interventions and/or lab practicals.

Scoring:

- Score 0 = the dog walks smoothly into the room (relaxed posture, tail base is not tucked), seems interested in the environment
- Score 1 = the dog is hesitant (may briefly stop and/or have lower body posture), but when the handler enters, the dog follows
- Score 2 = The dog is clearly scared when entering and/or pulls away from the room

8) **Handling (in a room on an examination table as described in g)** (Adapted from Valsecchi et al. 2011 and Åkerberg et al. 2012)

The tester calls the dog to him/her. He/she then picks up the dog and lifts it onto an examination table. Immediately offer the dog a piece of kibble: show it to the dog and place it on the table; do not feed it directly from your hand – wait 5 seconds, while making sure the dog does not jump off the table but do not interact with the dog otherwise. If the dog ignores the piece of kibble, pick it up again, show it to the dog and put it back down. Wait another 5 seconds. If the dog does not eat the kibble, take the piece of food away.

Next the tester performs a brief physical examination of the dog by stroking with both hands along the dog's sides to its hind legs, and looking into its ears and mouth. Finally, the dog is placed back on the ground and another piece of kibble is offered by placing it on the ground in front of the dog. Wait 5 seconds. If the dog ignores the kibble, pick it up again, show it to the dog (you can say the dog's name to get its attention), and place it back down. Wait another 5 seconds. Remove the kibble if it is not eaten.

Scoring part 1:

- Score 0 = calm or confident with human contact
- Score 1 = the dog shows mild stress signalling (green zone of ladder) and/or some freezing
- Score 2 = the dog shows intermediate stress signalling (orange zone of ladder) and/or freezing for more than half of the examination
- Score 3 = the dog freezes for the entire examination
- Score 4 = the dog responds aggressively (red zone of ladder)

Green zone: yawning, blinking, lip licking, turning head away, turning body away, sitting down, lifting a front paw
Orange zone: walking away, low posture, ears back, maintaining a crouched position, tail tucked
Red zone: Stiffening and staring, growling, snapping, biting

Scoring part 2:

- Score 0 = The dog eats both treats within 15 seconds
- Score 1a = The dog eats only the first treat within 15 seconds
- Score 1b = The dog eats only the second treat within 15 seconds
- Score 2 = The dog does not eat any of the treats within 15 seconds

The tester now walks the dog back to the animal house, but remains in a quiet area. The dog is allowed a few minutes to explore this area.

9) **Attention test / tracking** (adapted from Overall, pers. Comm and Valsecchi et al. 2011)

The dog is in back in the animal house, in a quiet area (where a crate is already present). It is still on a lead and may be sitting or standing for this test. It is most convenient if a handler holds the lead and the tester places him/herself in front of the dog. The tester shows the dog a piece of kibble (hold it close enough for the dog to sniff it), then closes the hand, holds it at about 0,5m from the dog and moves the hand in front of the dog from left to right for 10 seconds.

Scoring:

- Score 0 = the dog holds the gaze continuously for at least three of the 10 seconds
- Score 1 = the dog holds the gaze intermittently
- Score 2 = dog does not hold the gaze and/or walks away
- Score 4 = eyes flick / strabismus

10) **Crating**

The dog remains in the same room as the attention test. Let the dog off the leash. Walk to the crate, open the door of the crate and encourage the dog to go in (max. 2 seconds encouragement – <u>do not</u> use a treat to lure the dog!). Wait 5 seconds. If the dog did not go in, repeat the encouragement for 2 seconds. Wait another 5 seconds. If the dog enters the create, it is rewarded.

Scoring:

- Score 0 = goes in readily
- Score 1 = goes in only with encouragement
- Score 2 = the dog does not enter the crate voluntarily (or only enters the crate partially and walks away again)

11) **Stuffed Kong**

With the dog still off leash and watching you (call the dog over if needed), put down a Kong holding 5 pieces of kibble in the crate. Step back (2 m). Record the time for the dog to approach the Kong close enough to touch it (maximum time of 5 minutes). Record whether the dog attempts to get the food. Score the timing and behaviour.

Scoring part 1: timing:

- Score 0 = approaches Kong within 2 seconds
- Score 1 = approaches Kong within 10 seconds
- Score 2 = does not approach the Kong

Scoring part 2: retrieving food:

- Score 0 = retrieves at least one piece of kibble from the Kong
- Score 1 = attempts to retrieve kibble, but stops before getting one piece out of the Kong
- Score 2 = sniffs the Kong, but does not attempt to retrieve the kibble

Scoring part 3: emotion:

- Score 0 = does not show signs of hesitation/fear
- Score 1 = shows signs of hesitation/fear but these improve over time
- Score 2 = shows signs of hesitation/fear but they do not improve (remain of similar frequency/intensity or get worse)

When this subtest is done for the first time, it should be noted whether the dog is already familiar with a Kong.

12) Return to and entering kennel (scoring adapted from Valsecchi et al. 2011)

a. Return to kennel

Put the leash on the dog and walk back to the kennel room.

Scoring:

- Score 0 = the dog walks smoothly through the corridor (relaxed posture, tail base is not tucked), seems interested in the environment
- Score 1 = the dog is hesitant (may have lower body posture and tail base is possibly tucked), but when the handler walks, the dog follows and it does not stop intermittently
- Score 2 = The dog walks with the handler, but stops intermittently (by sitting or lying down without sniffing) during walking
- Score 3 = The dog is clearly scared (flattens itself when walking or stopping) and/or pulls strongly towards the animal house/kennel

b. Entering the kennel

When in the animal room, close the door and let the dog off leash. Throw treats into the kennel to keep the other dogs from escaping. Open the kennel door. Throw in a few more treats.

Scoring:

- Score 0 = the dog enters the kennel immediately
- Score 1 = the dog is reluctant to enter
- Score 2 = the dog refuses to enter and pulls back strongly
