# Supplementary material for: Network analysis reveals abnormal functional brain circuitry in anxious dogs
Source: PLoS One. 2023 Mar 15;18(3):e0282087. doi: 10.1371/journal.pone.0282087 (PMC10016658; doi:10.1371/journal.pone.0282087)
Supplement: S1 Fig — (DOCX) [file pone.0282087.s002.docx]

**Supplemental material**

| 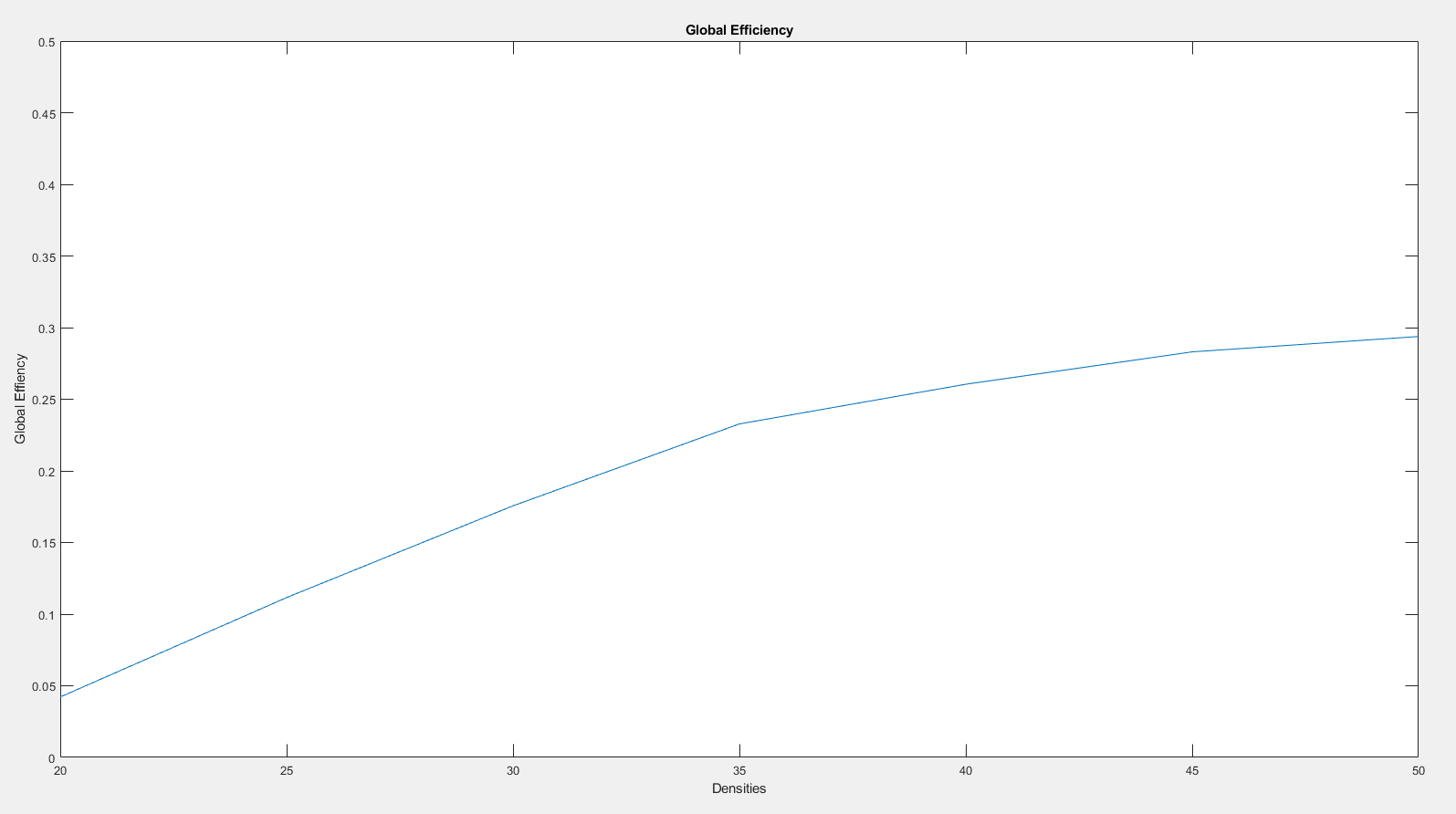  a |
| --- |
| 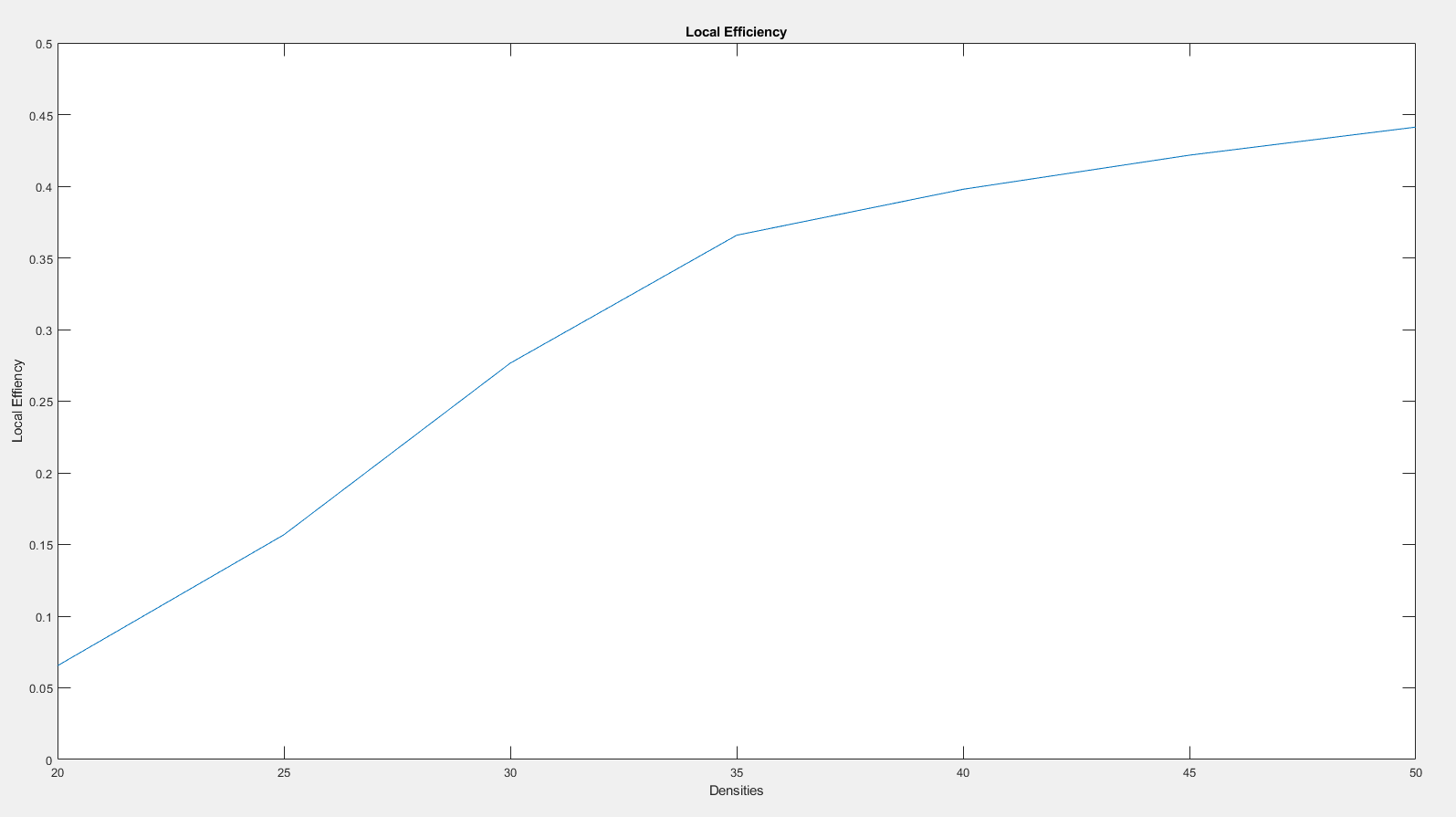  b |
| 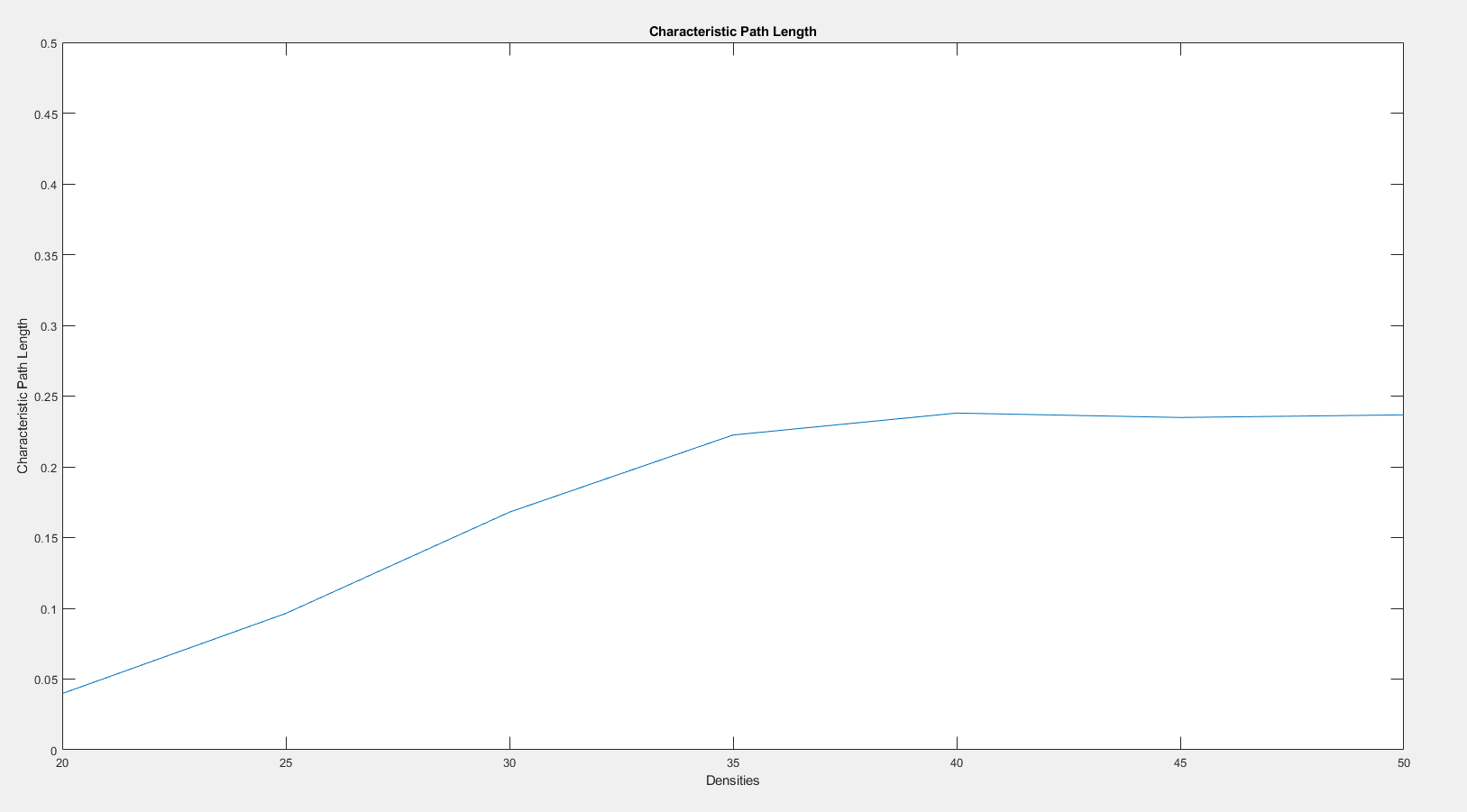  c  Fig. S1 The global efficiency (a), local efficiency (b), and characteristic path length (c) of amygdala in a range of sparsity thresholds (20%-50%, with 5% intervals) |
