# Supplementary material for: Network analysis reveals abnormal functional brain circuitry in anxious dogs
Source: PLoS One. 2023 Mar 15;18(3):e0282087. doi: 10.1371/journal.pone.0282087 (PMC10016658; doi:10.1371/journal.pone.0282087)
Supplement: Attachment — Submitted filename: response to reviewers.docx [file pone.0282087.s003.docx]

**Comments from Reviewer 1**

Main:

1) The authors picked 5 brain regions to study the ‘anxiety circuit’. It would be good to include a rationale for including these regions.

Also, ‘frontal lobe’ is rather unspecific, as both rodent and human work indicates subregional specificity within the frontal cortex in the modulation of anxiety, with some subregions boosting and others suppressing anxiety. Why did the authors decide to take this rather unspecific readout?

We thank the referee for these insightful comments.

First, we picked these five regions based on our former research in behavior-disordered dogs, and the known human counterparts, since no clear information is available regarding the anxiety circuitry in dogs. To clarify, we have added following information in the method section:

Line 234-237: Based on our former research [8, 13, 26, 27, 38-40], relevant veterinary behavior medicine research [41], and human medicine [28-31], we choose these five regions of interest as the anxiety circuit in this study: amygdala, frontal lobe, hippocampus, mesencephalon, and thalamus.

For the ‘frontal lobe’, however, we must mention that, in contrast to rodents, in the anxiety canine model clearly showing behavior abnormalities little is known about specific anxiety subcircuits in the frontal cortical areas. Therefore, we had decided to not subdivide the frontal cortex, given that the frontal cortex by itself is not the focus of this research. Nevertheless, we acknowledge this as a study limitation, so we have added the following sentence to the discussion section:

Line 458-461: Fourth, that we have included the entire prefrontal as ROI should be considered as a limitation, given that this is a large region including subregions with potentially different functionalities in the dog anxiety system, although this is at this moment not yet established in the dog.

2) Furthermore, the data don’t really convince me about the specificity of the reported effects to the anxiety circuit. Overall, effects seen in nodal network metrics are also found on the global level, but there they only fail to reach significance. As the authors run tests on all of the brain regions independent of each other, the effects on these regions are not directly compared, making that specificity cannot be claimed. Would there be a ‘control’ region that could be taken along to compare the effects against? Or could the authors maybe include all regions as within subject factor, to test whether all regions were similarly affected or whether effects were region-specific? The effects in the amygdala at least seem to be robust, and differ in same cases (clustering coefficient) from the other regions?

Fair points.

As requested, we added vermis as ‘control’ region in the nodal level analysis. Therefore, between-group differences in the nodal level were assessed in the regions of anxiety circuit (including the frontal lobe, hippocampus, thalamus, amygdala, and mesencephalon), and we added the vermis as a ‘control’ region using non-parametric Mann-Whitney U test. To correct the network parameters for multiple comparisons, we applied FDR correction by using p < .05 as the significant threshold. We found that the patient group exhibited significantly increased global efficiency and local efficiency in the amygdala (p = 0.007, FDR correction; p = 0.003, FDR correction, respectively), compared with the control group. We also found that the patient group exhibited significantly decreased characteristic path length in the amygdala (p = 0.006, FDR correction), compared with the control group.

Consequently, we have revised the manuscript accordingly with these more stringent corrections.

In method section:

Line 237-243: The vermis was chosen as the control region. Therefore, between-group differences in the nodal level were assessed in the regions of the anxiety circuit and vermis. The nonparametric Mann-Whitney U test was performed in the nodal level analysis to compare the between-group differences. To correct the network parameters for multiple comparisons, false discovery rate (FDR) correction was applied by using p < .05 as the significant threshold.

In results section:

Line 288-292: In the amygdala, we found that the patient group exhibited significantly increased global efficiency and local efficiency (p = 0.007, FDR correction; p = 0.003, FDR correction, respectively), and significantly decreased characteristic path length (p = 0.006, FDR correction), compared with the control group.

3) Related to point 2, is the number of comparisons made by the authors and the corrections for multiple testing that are actually performed. Whereas the authors do report on FDR correction for connection readouts and connectivity correlations, they do not correct any of the other readouts for the comparisons made. For example Fig 3B only already contains 25 comparisons, and none of them seems corrected for multiple testing. Similarly, Fig 5 reports on significant correlations that are not corrected for multiple testing, except for the connectivity readout.

Thank you for this remark. Normally in graph analysis, we only corrected for multiple comparisons between regions, not between parameters. As we answered in point 2, at nodal level, we found that only in the amygdala, the global efficiency, local efficiency and characteristic path length were significantly different between patient and control groups. If we corrected for 25 comparisons, there were no significantly difference.

Minor:

4) It is not completely clear to me what the exact rationale is for the study. Is it to better understand and treat anxiety in dogs, or to develop an animal model for anxious patients to allow for more invasive recordings/manipulations? The latter is mainly done in rodents, and if the authors think their model is superior to this, it would be good to further explain their reasoning. Currently, the MS includes both an introduction on human anxiety and that in dogs; the work might benefit from making the goal very clear and tailor the introduction and discussion towards this goal.

The goal of this study is bifold: anxiety in dogs and natural animal model. Rodent model research is okay but does not provide a natural model as in general it is conducted on genetical, pharmacological, physical, manipulated animals.

Consequently, we have adapted some parts in the introduction and discussion.

In introduction section:

Line 69-71: Thus, dogs might be a better model to investigate the mechanisms of anxiety as the rodent research is in general conducted on genetical, pharmacological, physical, manipulated animals.

Line 115-117: The results in dogs will benefit both veterinary medicine for anxiety-disordered animals and may serve human medicine as a natural model.

In conclusion section:

Line 469-472: Such efforts will provide important insight in pathophysiological mechanisms and anxiety illness course in dogs that may lead to more personalized and effective therapies and provide a natural animal model for human medicine.

5) The manuscript would benefit from including a brief explanation of the distinct readouts of graph theory in a layman’s style in the introduction or results section. Terminology such as global efficiency, path length and nodal degree is difficult to grasp for non-experts, whereas the findings might also be of interest to them.

Agreed. We have added some information in the introduction section about graph theory parameters, and Table 3.

Line 103-109: Specifically, nodal degree measures the degree of nodes tending to cluster together, global efficiency measures the efficiency of parallel information transfer through the network, clustering coefficient measures the efficiency of information exchange within a local subnetwork or among adjacent regions, characteristic path length measures the ability for information propagation within the network, the small-worldness indicates a typical network that has similar path length but higher clustering than a random network [18-20].

Line 243-244: The calculation of network measures of integration, segregation and centrality is summarized in Table 3 [18].

**Comments from Reviewer 2**

1 Did the Authors perform field inhomogeneity-related artifact correction?

Thank you for this point. We didn’t perform field inhomogeneity-related artifact correction since this is not typically done during preprocessing of rsfMRI. However, we did perform 2^nd^ order shimming before image acquisition to optimize the field homogeneity. This point has been added to the revision.

2 Were the global WM and CSF signal regressed in fMRI processing?

Good point. We did not do global WM and CSF signal regression. Regression of these signals is considered controversial, with some researchers believing that it leads to loss of information [26]. Because of this controversy, we decided not to regress out global WM and CSF signals. This point has been added to the revision.

[26] Grajauskas, L. A., Frizzell, T., Song, X., & D’Arcy, R. C. (2019). White matter fMRI activation cannot be treated as a nuisance regressor: Overcoming a historical blind spot. Frontiers in neuroscience, 13, 1024.

3 Did the Authors check whether the brain connectomes exhibit a small-world behavior in line with current knowledge on brain networks? This should be verified considering the relative low number of nodes in the networks

Thank you for this remark. The small-world metrics normalized clustering coefficient, normalized characteristic path length and small-worldness are similar to those of the macaque and mouse brain, that exhibit small-world behavior [19]. We found σ > 1 for the two groups, indicating that both patient and control groups exhibited small-world. Thus, we have added the point in the revision:

Line 230-232: Small-worldness (σ) indicates a typical network that has similar path length but higher clustering than a random network [19].

Line 279-281: In addition, we found σ > 1 for the two groups, indicating that both patient and control groups exhibited small-world attributes.

[19] Bassett, D. S., & Bullmore, E. T. (2017). Small-world brain networks revisited. The Neuroscientist, 23(5), 499-516.

4 How did they deal with negative weights in brain connectomes?

Only positive weights were included, negative weights were disregarded, as is typically done in graph theoretical analyses.

5 Have they checked if there are disconnected nodes in the functional connectomes after the thresholding procedure?

It was checked. All nodes have a degree higher than 1, so no disconnected nodes, although we still might have disconnected clusters.

6 Did the authors compute the network measures on the weighted or binarized networks?

Thanks for this point. We computed the network measures on the weighted networks. To make it clear, we have added this point in the revision.

7 P.11, line 204, degree and strength are two different measures. Did the authors compute the degree or strength? Or both? Throughout the manuscript they refer to the degree.

We computed the strength. We adapted this sentence in the revision as:

Connection strength is the number of (weighted) edges connected to a node.

8 More details, formula and related references should be reported for the computation of the network measures

We have added a new table describing the computation of the network measures in the method section.

9 Did they compute the network measures for all the nodes or only those involved in the anxiety circuit? This should be better clarified in the methods section.

We computed the network measures for all the nodes. As requested, we added this into the revision.

For remarks 1,2, 4, 6, and 9, we have added these points in the method part:

Line 194-197:

During the processing, field inhomogeneity-related artifact correction was not performed, global white matter (WM) and cerebrospinal fluid (CSF) were not regressed [26], only positive weights in brain connectomes were included, and the network measures for all the nodes were computed on the weighted networks.

10 Did the authors take into account multiple comparisons correction for the node-level analysis? Are the reported -pvalues uncorrected? I feel that some form of correction is warranted to ensure that nodes exhibiting different nodal topology do not suffer from multiple statistical tests (across different nodes and across different nodal measures).

Yes, we had corrected for multiple comparisons between nodes in the anxiety circuit. We have clarified this better in the revision.

Line 241-243:

To correct the network parameters for multiple comparisons, FDR correction was applied by using p < .05 as the significant threshold.

11 Why did they average left and right in node-level analysis of the anxiety circuit? It would be interesting to assess whether hemispheric differences exist

We didn’t assess whether hemispheric differences exist in this study. According to a rat study of our team with similar processing procedures, changes were very similar left and right (Christiaen et al., 2019). The advantage is that if there are less comparisons, less stringent correction necessary.

CHRISTIAEN, E., GOOSSENS, M.-G., RAEDT, R., DESCAMPS, B., LARSEN, L. E., CRAEY, E., CARRETTE, E., VONCK, K., BOON, P. & VANHOVE, C. 2019. Alterations in the functional brain network in a rat model of epileptogenesis: A longitudinal resting state fMRI study. *Neuroimage,* 202**,** 116144.

12 In the conclusion section, the Authors state that “rs-fMRI could be used as a biomarker for anxiety”, I would suggest toning down this section as no conclusive evidence on this direction can be drawn from the present study. Correlation findings, especially on such a small sample, are not indicative of potential biomarkers.

Indeed, the former conclusion we made was a bit ambitious. We have adapted the conclusion section in this revision:

Line 465-469: As we found correlations between anxiety symptoms and network measures, this may indicate that rs-fMRI could provide useful diagnostic information for anxiety in dogs, although further research is still required. In the future, we would also like to investigate the potential of rs-fMRI as a diagnosis tool for treatment response, such as pharmacological treatments or neural modulation treatments like rTMS.
