# Supplementary material for: Network analysis reveals abnormal functional brain circuitry in anxious dogs
Source: PLoS One. 2023 Mar 15;18(3):e0282087. doi: 10.1371/journal.pone.0282087 (PMC10016658; doi:10.1371/journal.pone.0282087)
Supplement: Attachment — Submitted filename: Response to reviewers-20221027.docx [file pone.0282087.s004.docx]

**Reviewer #1:** The authors have answered most of my comments.

However, one of my previous points is not yet optimally dealt with:

"4) It is not completely clear to me what the exact rationale is for the study. Is it to better understand and treat anxiety in dogs, or to develop an animal model for anxious patients to allow for more invasive recordings/manipulations? The latter is mainly done in rodents, and if the authors think their model is superior to this, it would be good to further explain their reasoning. Currently, the MS includes both an introduction on human anxiety and that in dogs; the work might benefit from making the goal very clear and tailor the introduction and discussion towards this goal."

The authors state in their answer that their goal is bifold, anxiety in dogs and natural animal model. I totally agree on anxiety in dogs, but the statements on rodent research are rather blunt. Rodents may be evolutionary more distinct from humans than dogs, but the manipulations listed are not always implemented, and if applied, serve specific purposes such as targeted manipulations of cells or circuits to study their effects on behavior. Studies in dogs have other flaws; for example, that there is much less knowledge to build on than in rodents, and there might be more stringent ethical restrictions to work in dogs (and less possibilities for manipulations to study causality). According to the 3R principle the work in dogs should have clear benefits over working with other animal models, to warrant their use. As such, I am not convinced by the current answer and text added to the MS. A more nuanced discussion would benefit the paper.

Thank you for this useful remark. In fact, the realm of our research, is all that. It is to better understand and treat anxiety in dogs and develop an animal model. However, the referee is right that putting it all here in this manuscript is confusing, and it does not help the clarity of the paper. Of course, in this paper the goal is not to prove that dog models are superior to rodent models, as we did not include rodents in our sample.

We agree with the referee in stating that rodent models can serve better in specific purposes. We only hypothesize, that our canine model can serve as a more natural translational model, together with rodent and other animal models, which can be model for for human anxiety (and vice versa). So, we have toned town our former statement as followed:

Introduction part:

Line 70-72: Thus, the canine species might be an appropriate model to investigate brain networks involved in anxiety, and together with other animal research, such as rodents, can be used as a model for human anxiety (and vice versa).

Discussion part:

Line 470-473: Such efforts will provide important insight into pathophysiological mechanisms of anxiety in dogs, which can lead to more personalized and effective therapies, and together with other animal research, build a bridge to the understanding of human behavior (and vice versa).

**Reviewer #2:** I thank the Authors for addressing most of my concerns. I have a few comments yet.

1. Was the C-BARQ administered both to healthy and anxious dogs?

No, the C-BARQ was only administered to anxious dogs as mentioned in M&M-Patient recruitment. The healthy beagles got regular check by the caretakers and veterinarians, as mentioned in M&M-Animal.

2. Lines 196-197, I do not understand way the Authors chose to report “only positive weights in brain connectomes were included, and the network measures for all the nodes were computed on the weighted networks.” in the Preprocessing section. This explanation would better fit the section Functional network construction.

Good point. We have moved this to the Functional network construction section in line 208-209.

3. Did the Authors check whether the network measures were consistent across the range of thresholds explored?

No, we didn’t check. But probably the network measures were proportional to network density because of the weighted edges, as mentioned in M&M Functional network reconstruction.

4. Line 258, should “connections” read “comparisons”?

Thank you for this point. Yes, it should be “comparisons”. We have adapted it in the manuscript.

5. Was the statistical analysis at the connection level performed on the 30x30 connectivity matrix? Or did the authors extract only the connectivity patterns between the five regions of interest? Reading lines 295-296, I assume the latter. In this case, why did the Authors choose to create 30x30 connectivity matrices instead of 5x5 matrices? What is the role of the remaining brain regions? In addition, by doing this, the node-level topological measures reflect not only the of connectivity between the anxiety-related regions, but also the connectivity patterns of such regions with the remaining 25 brain areas. And this may also explain why no changes in global network topology have been identified.

Fair point. We wanted to look at the effect of anxiety on the whole brain and investigate whether whole-brain connections of the anxiety regions were affected, not just the connections in the anxiety circuit. For our graph theory application, the selected ROIs do not necessarily respect the functional boundaries of the brain ^[1, 2]^. We did 30x30 because we assumed they are involved in anxiety, but we didn’t find global level difference, so, based on the calculated results from 30x30, we selected these 5 key regions for further analysis. On the other hand, if we would have chosen the connectivity matrices 5x5, the rest regions (25 regions) would not have been involved. This would have excluded the rest regions as potential influential nodes in the network.

[1] Yu Q, Du Y, Chen J, et al. Application of graph theory to assess static and dynamic brain connectivity: Approaches for building brain graphs[J]. Proceedings of the IEEE, 2018, 106(5): 886-906.

[2] Khullar S, Michael A M, Cahill N D, et al. ICA-fNORM: Spatial normalization of fMRI data using intrinsic group-ICA networks[J]. Frontiers in systems neuroscience, 2011, 5: 93.
