# Supplementary material for: Network analysis reveals abnormal functional brain circuitry in anxious dogs
Source: PLoS One. 2023 Mar 15;18(3):e0282087. doi: 10.1371/journal.pone.0282087 (PMC10016658; doi:10.1371/journal.pone.0282087)
Supplement: Attachment — Submitted filename: Response to reviewers20230130.docx [file pone.0282087.s005.docx]

**Response to reviewers:**

We would like to thank the reviewers again for their very helpful and detailed comments.

**Reviewer #2:**

6. Review Comments to the Author

Reviewer #2: The Authors have answered most of my comments.

However, one of their answers to one of my previous comments is not clear to me.

“3. Did the Authors check whether the network measures were consistent across the range of thresholds explored?

In the main text the Authors state that “Network metrics were calculated at different correlation matrix densities, from 20% to 50% density with a 5% interval, and averaged over these densities”. If they computed the metrics why they did not check for network measure consistency across the range of thresholds? They may easily check it by plotting network measure values against thresholds. While not emerging from the text that the network measures were proportional to network density because of the weighted edges, the fact that some of the network measures may be proportional to network density does not necessarily means that the topology of the network is stable across the thresholds. In addition, could the Authors explain how they chose the specific density range 20-50%?

Thank you for this important comment.

The reason why we chose to specify the density range to be 20-50% is because the typical range for densities is 5-50% (standard in GRETNA), and because it is a balance between including enough regions to maintain a detailed representation of the brain while excluding noise or irrelevant voxels that could interfere with the analysis. But it is not a fixed standard [1, 2]. In our study, we did not include the lowest densities because there were no connections at the lowest densities, as indicated in the manuscript line 209-212 “To remove the weakest connections, thresholds based on network density (i.e., the number of remaining connections divided by the maximum number of possible connections) were applied to the correlation matrices.” We chose to start from 20% since then it is possible to compute network measures for this density and wanted to keep as close to the approved range by GRETNA.

And we agree with the reviewer that some of the network measures may be proportional to network density does not necessarily mean that the topology of the network is stable across the thresholds. Thus, we did the check for the consistency of the network measures across the range of thresholds in amygdala (these are the network measures described in our article), the results were shown below.

a

b

c

Fig. S1 The global efficiency (a), local efficiency (b), and characteristic path length (c) of amygdala in a range of sparsity thresholds (20%-50%, with 5% intervals)

As depicted in Fig.S1, even at low densities (20%), connections related to the amygdala are present in the surviving networks (5 regions) of 2 connections. This indicates that connectivity related to the amygdala is high and likely crucial. And starting at 35% densities, the results indicate a stable trend, which is convincing that the network measures are highly stable. In brief, the network measure consistency is stable across the range of 20% to 50% thresholds.

We added the consistency analysis results into the Supplement material and mentioned in the revised manuscript.

1.	Rubinov M, Sporns O. Complex network measures of brain connectivity: Uses and interpretations. NeuroImage. 2010;52(3):1059-69. doi: <https://doi.org/10.1016/j.neuroimage.2009.10.003>.

2.	Hallquist MN, Hillary FG. Graph theory approaches to functional network organization in brain disorders: A critique for a brave new small-world. Netw Neurosci. 2019;3(1):1-26. Epub 2019/02/23. doi: 10.1162/netn_a_00054. PubMed PMID: 30793071; PubMed Central PMCID: PMCPMC6326733.
